# Assessment of Deterioration Characteristics of Solid Waste-Based Binder-Stabilized Laterite Under Multiple Environmental Factors Based on Micro-Drilling Resistance

**DOI:** 10.3390/ma19194112

**Published:** 2026-09-25

**Authors:** Bing Yue, Qiong Zhang, Jiaxing Hu, Shengli Zhu, Wei Qiao, Haitao Li

**Affiliations:** 1 School of Smart City Engineering, Anyang Normal University, Anyang 455000, China; yue@email.cugb.edu.cn; 2 Henan Province Engineering Technology Research Center of Digital-Intelligent Building and Low-Carbon Building Materials, Anyang Normal University, Anyang 455000, China; 3 School of Engineering and Technology, China University of Geosciences (Beijing), Beijing 100083, China; 2102240132@email.cugb.edu.cn (S.Z.); 3102230001@email.cugb.edu.cn (W.Q.); 4 State Key Laboratory of Lithospheric and Environmental Coevolution, Institute of Geology and Geophysics, Chinese Academy of Sciences, Beijing 100029, China; zhangqiong@mail.iggcas.ac.cn; 5 Aviation Industry Corporation of China Institute of Geotechnical Engineering Co., Ltd., Beijing 100191, China; hujx046@avic.com

**Keywords:** solid waste-based binder, stabilized laterite, drilling resistance measurement system, response surface methodology, deterioration mechanism

## Abstract

Solid waste-based binder-stabilized laterite is prone to progressive deterioration under multiple coupled environmental factors in practical engineering applications. Conventional compression and direct shear tests fail to accurately characterize the gradient deterioration pattern of stabilized laterite specimens. In this study, a multi-factor test scheme was designed using response surface methodology, with wet–dry cycle number, initial humic acid content, and magnesium sulfate solution concentration selected as environmental factors. Continuous drilling resistance–drilling depth curves of stabilized laterite specimens were obtained using a drilling resistance measurement system, thereby quantitatively determining the deterioration depth and indirectly characterizing structural integrity via the drilling resistance in the stable drilling stage. Scanning electron microscopy observations were used to reveal the microscopic deterioration mechanism of stabilized laterite under the effects of environmental factors. The results of the regression show that the micro-drilling resistance in the stable drilling stage of undeteriorated stabilized laterite has a significant linear correlation with shear strength, indicating that micro-drilling resistance, as an indirect index of specimen integrity, also has potential for application in evaluating the strength of stabilized laterite. All three environmental factors increase the deterioration depth of stabilized laterite; wet–dry cycles exert the most significant effect and exhibit a statistically significant interaction within the investigated experimental domain with humic acid content, followed by magnesium sulfate concentration. For stabilized laterite subjected to wet–dry cycles and sulfate attacks, there exists a change point depth on the drilling resistance curve. Below this depth, the average micro-drilling resistance of the interior soil is mainly governed by the initial humic acid content. Microscopically, humic acid particles form weakly cemented zones in surrounding areas, which gradually evolve into preferential flow paths under wet–dry cycles, accelerating water infiltration and expanding deterioration depth. Magnesium sulfate crystallizes within pores to generate ettringite and other products, inducing extrusion stress on the cemented structure and further aggravating soil loosening and microcrack propagation.

## 1. Introduction

Soil modification is a widely adopted geotechnical improvement technique. It improves the engineering performance of weak soils by adding binders that modify the physical and chemical properties of the soil. Steel slag, fly ash, and blast furnace slag are typical industrial solid wastes generated from the steel and thermal power industries [1,2]. These materials are rich in active silicon, aluminum, and calcium components and contain abundant metastable glassy structures [3,4,5]. A solid waste-based binder (SWBB) fabricated by mixing the three wastes via alkali activation can undergo dissolution, depolymerization, and polymerization reactions after blending with soil, forming a hardened cementitious matrix with satisfactory mechanical strength and durability [6]. Accordingly, SWBB can serve as an alternative to traditional Portland cement for soil stabilization. In practical engineering service, stabilized soils inevitably suffer from environmental deterioration. Specifically, stabilized soil may endure periodic wet–dry cycles induced by alternating rainfall and evaporation [7]. Additionally, for stabilized laterite with high initial humic acid content, anions dissociated from humic acid under alkaline conditions can combine with metal cations released from the SWBB to form stable complexes and insoluble precipitates, reducing the yield of hydration products [8]. For soil stabilization projects near high-sulfur mining areas, the high sulfate concentration in surface and groundwater facilitates the formation of poorly cemented or non-cementitious crystalline minerals within stabilized soil. The resultant expansion stress damages soil integrity and causes strength deterioration [9].

Under the combined effects of wet–dry cycles and external sulfate attacks, SWBB-stabilized soil exhibits a progressive structural deterioration from the surface inward [10]. Under the influence of this structural deterioration, both the structural integrity and strength of the specimen will be affected. In existing studies, the assessment of specimen deterioration is usually achieved by preparing standard specimens (e.g., ASTM D2166 [11] requires cylindrical specimens with a height-to-diameter ratio between 2 and 2.5), subjecting them to deterioration, and comparing the mechanical test results of the specimens before and after deterioration. This test method is simple and intuitive, but it can only reflect the overall average mechanical properties of a specimen and cannot accurately characterize the gradient deterioration behavior of the surface layer. The drilling resistance measurement system (DRMS) drives a miniature drill bit to penetrate stabilized soil specimens at a constant velocity. It continuously records drilling resistance and penetration depth during testing [12,13,14], acquiring full-scale resistance data from the specimen surface to the interior. The variation in micro-drilling resistance can effectively reflect the degree of integrity weakening of the structure along the penetration depth [15]. Through fitting analysis, it is also possible to examine whether micro-drilling resistance is correlated with mechanical properties. This enables the micro-drilling resistance value not only to serve as an indicator of specimen integrity but also to provide a reference for comparing the mechanical properties of specimens.

Response surface methodology (RSM) has been widely applied to investigate cement-based cementitious materials by modeling multi-variable interactive relationships [16,17]. In previous durability studies on concrete subjected to complex environmental conditions, RSM has been utilized to establish quantitative correlations between environmental factors and material strength evolution [18]. Similarly, this method is also applicable to exploring the deterioration characteristics of SWBB-stabilized soil under environmental coupling effects. It enables a rigorous comparison of the influence magnitude of individual environmental factors by clarifying the quantitative relationship between deterioration behavior and external environmental conditions.

In this study, the DRMS was used to test the micro-drilling resistance of both undeteriorated stabilized laterite specimens and stabilized laterite specimens exposed to different environmental conditions. Based on the measured micro-drilling resistance–drilling depth curves, the deterioration depth of the specimens was determined from the stage-wise variation characteristics of the drilling resistance. Micro-drilling resistance was used as an indirect index of the integrity of the stabilized laterite specimens, and a fitting analysis was conducted between the stable-drilling-stage micro-drilling resistance and the strength properties of the specimens to investigate whether a correlation exists between these two variables. This study employed RSM to design multi-factor experiments and investigate the coupled effects of multiple environmental factors on SWBB-stabilized laterite. In the RSM experiment, the deterioration depth and stable-drilling-stage micro-drilling resistance of stabilized laterite under environmental exposure were taken as response variables, and quadratic polynomial regression fitting was performed for the two evaluation indices. Analysis of variance (ANOVA) was conducted to quantify the significance of each term in the regression models. The influencing mechanisms of the number of wet–dry cycles, initial humic acid content, and magnesium sulfate concentration on the deterioration depth and stable-drilling-stage micro-drilling resistance of stabilized laterite were systematically compared and analyzed. Finally, by combining these results with micro-morphological observations, the microscopic deterioration mechanisms of stabilized laterite under multiple coupled environmental factors were revealed.

## 2. Materials and Methods

### 2.1. Test Raw Materials

The raw materials used in this study include laterite collected from Nanchang City in the middle and lower reaches of the Yangtze River, China, three types of industrial solid wastes (steel slag from a steel plant in Shijiazhuang, Hebei Province; fly ash from a coal-fired power plant in Zhengzhou, Henan Province; and blast furnace slag from a slag yard in Xinxiang, Henan Province), as well as sodium hydroxide (NaOH) solution adopted as the alkali activator.The NaOH solid particles for solution preparation were purchased from Sinopharm Group, Beijing, China. The SWBB, formulated by combining the three solid waste materials with a NaOH solution, was mixed into laterite to prepare modified-soil specimens. The laterite used in this study is an iron- and aluminum-rich cohesive soil formed by intense laterization in subtropical regions and exhibits good mechanical properties under low-moisture conditions; however, as the moisture content increases, its plasticity increases significantly [19] and its strength drops sharply [20,21]. After testing, the natural density of the laterite was determined to be 1.63 g/cm^3^. Atterberg limits were determined according to ASTM D4318 [22] on the <425 µm fraction. The liquid limit was measured by the Casagrande multipoint method (25 blows), and the plastic limit by the 3.2 mm thread method. The results indicate a liquid limit of 47.7%, a plastic limit of 18.1%, and a plasticity index of 29.6%. Its macro-morphology before stabilization and the microscopic images obtained by SEM observation are shown in Figure 1. A heating treatment was applied to pulverized laterite to eliminate trace native humic acid within the raw material, so as to avoid interference with the follow-up experimental results. The detailed heating procedure is described as follows. The pulverized laterite sieve was placed in an oven and heated at 150 °C for 24 h. At the adopted temperature and holding duration, only adsorbed water and loosely bound humic substances are removed. This low-temperature condition cannot trigger clay mineral dehydroxylation or mineral phase transformation.

The microscopic morphology images of laterite obtained by SEM observation reveal that the main constituents of laterite are relatively large quartz particles, interspersed with layered and flaky clay mineral particles. Due to their large specific surface area and negatively charged surfaces, the clay particles in laterite adsorb polar water molecules through electrostatic attraction, forming a bound water film on the particle surfaces. The thickness of this bound water film varies with the moisture content of the soil [23], which is manifested macroscopically as significant variations in the shear strength parameters of laterite at different moisture contents. The cohesion and internal friction angle at different moisture contents obtained from direct shear tests are shown in Table 1.

A laser particle size analyzer(Winner2000ZD, Jinan Winner Particle Instrument Stock Co., Ltd., Jinan, China) was used to conduct particle size tests on oven-dried and ground laterite, and the test results are presented in Figure 2.

Steel slag and blast furnace slag were pre-processed into powder, and the fly ash is Class F fly ash. The particle size distributions of the three solid waste materials are also shown in Figure 2.

The main oxide mass fractions of laterite and the three solid waste materials determined by XRF tests are shown in Figure 3.

XRF results show that steel slag and blast furnace slag possess high calcium content, while fly ash is rich in silicon and aluminum. Based on this chemical characteristic, blending the three materials can realize component complementarity, which is favorable for pozzolanic reaction.

In a previous study [24], the formulation of the SWBB suitable for stabilizing laterite was determined as follows: steel slag, fly ash, and blast furnace slag were uniformly mixed in a mass ratio of 1.71:1.27:1 to prepare the solid waste mixture. Then, a NaOH solution at a concentration of 5 mol/L was added to the solid waste mixture (the mass ratio of NaOH solution to solid waste mixture was 0.38) and stirred evenly to produce the SWBB. In practical application, the dosage of the SWBB is controlled based on the principle that the mass of the solid waste mixture accounts for 22.3% of the dry mass of laterite. After stabilization with this SWBB and 7 days of curing, the stabilized laterite can still achieve a shear strength of over 500 kPa even when saturated with water, demonstrating that the SWBB has a favorable stabilization effect. Based on existing work concerning SWBB-stabilized laterite, this study further explores the deterioration characteristics of SWBB-stabilized laterite under the combined action of multiple environmental factors using the micro-drilling resistance test method.

### 2.2. Specimen Preparation Method

For SWBB-stabilized laterite, sufficient internal hydration reactions produce abundant hydration products, thereby forming a denser cementitious structure. A denser matrix requires greater drilling penetration resistance, corresponding to higher micro-drilling resistance values. Notably, micro-drilling resistance cannot directly represent soil strength; thus, correlation verification between micro-drilling resistance and mechanical parameters is essential to validate its feasibility as an indirect strength index [25]. Accordingly, prior to environmental deterioration tests, undeteriorated stabilized laterite specimens with curing ages of 3 d, 5 d, 7 d, 14 d, 21 d, and 28 d were prepared. Their shear strength parameters and micro-drilling resistance data were separately tested, and a correlation analysis was subsequently conducted. In this study, the shear strength of undeteriorated stabilized laterite specimens at different curing ages was measured under both dry and saturated conditions. Meanwhile, the micro-drilling resistance of deteriorated specimens subjected to the coupled action of multiple environmental factors was tested. Fitting analyses were performed between micro-drilling resistance and shear strength (dry-state and saturated-state, respectively) of undeteriorated stabilized laterite specimens to explore their correlations and verify whether micro-drilling resistance can be adopted to indirectly characterize the strength properties of stabilized laterite.

When preparing the six curing age groups of undeteriorated SWBB-stabilized laterite specimens with curing ages of 3 d, 5 d, 7 d, 14 d, 21 d, and 28 d, the required dry powdered laterite was first weighed and spread evenly in a stainless steel tray. Subsequently, the moisture content of the laterite was increased to 15% using a spray bottle, then sealed and left to stand for 12 h. The moisture-conditioned laterite was then thoroughly mixed with the corresponding amount of SWBB. The resulting SWBB–laterite mixture was placed into a ring mold with an inner diameter of 80 mm and compacted using static compaction, so that the compacted stabilized laterite specimen had a thickness of 20 mm and a compaction degree controlled at 93%. Each curing age group contained five parallel specimens, of which two were used to test the shear strength after drying, two for testing the shear strength after saturation, and one for testing the micro-drilling resistance. After compaction, the specimens were placed in a standard curing chamber. Upon reaching the designated curing age, the specimens were taken out; for each group, three specimens were dried, and two specimens were saturated in a vacuum saturation device. The shear strengths of the two dried specimens and the two saturated specimens were tested separately and averaged to obtain the average shear strength of that group under dry and saturated conditions. The shear rate was set to 0.8 mm/min, and no normal stress was applied. Each test was terminated once the shear stress reached its peak value. For saturated specimens, vacuum saturation was performed prior to shearing. The one remaining dried specimen in the group was used for the micro-drilling resistance measurement.

Subsequently, the Box–Behnken design (BBD) was adopted to establish the response surface experimental scheme. Three influencing factors were selected: wet–dry cycle number, initial humic acid content of laterite, and magnesium sulfate concentration of the soaking solution used in wet–dry cycling. These three variables were denoted as A, B, and C, respectively. The magnesium sulfate concentration range was referenced from monitoring data of soils in comparable regional environments reported in previous studies [26]. The humic acid content range was established based on field-measured soil values from the relevant published literature [27]. Accordingly, the variation ranges for the investigated factors were defined as follows: 5–15 for wet–dry cycles, 0–5% for initial humic acid content, and 0–15 g/L for magnesium sulfate concentration. The high, medium, and low levels of each factor were coded as 1, 0, and −1, respectively. The coded and corresponding actual values for each influencing factor are summarized in Table 2.

According to the settings of the environmental influence conditions given in Table 2, the Box–Behnken design method was adopted to obtain the specific groupings of the SWBB-stabilized laterite specimens under the influence of different numbers of wet–dry cycles, soil humic acid contents, and magnesium sulfate solution concentrations, as shown in Table 3.

Based on the test conditions set out in Table 3, SWBB-stabilized laterite specimens were prepared and tested. Because the wet–dry cycling process spans a certain period of time and the continuous formation of hydration products during this process may significantly affect the integrity of the stabilized laterite, the curing age of the stabilized laterite specimens was set at 28 days. After the specimens reached this curing age, the hydration reaction had essentially entered a stable stage.

The SWBB-stabilized laterite specimens subjected to environmental factors had the same dimensions as the undeteriorated specimens, i.e., disk-shaped specimens with a diameter of 80 mm and a height of 20 mm.

The detailed specimen preparation and test procedures were as follows: The amount of laterite required for each group of specimens was calculated. According to the specimen grouping scheme given in Table 3, 15 portions of dry powdered laterite were weighed, and humic acid was added to each portion at the dosage specified in Table 3. Humic acid was incorporated in dry powder form. The dry humic acid powder was thoroughly dry-mixed with pulverized dry laterite in a mixer. This dry-mixing method mitigates powder agglomeration and ensures the uniform distribution of humic acid within the soil matrix. Subsequently, the moisture content of each soil portion was raised to 15% using a spray bottle. The soil samples were sealed and left to stand for 12 h. Afterward, the SWBB was prepared and added to each soil portion, followed by thorough mixing. The resulting SWBB–soil mixture was poured into a ring mold with an inner diameter of 80 mm and compacted by static compaction, so that the thickness of the compacted stabilized soil was 20 mm. After compaction, the specimens together with the molds were placed in a curing chamber for curing. Upon completion of curing, the specimens were removed from the chamber. Fifteen flat-bottomed containers were prepared, and a porous stone was placed at the bottom of each container. The ring mold holding the specimen was then placed on the porous stone, with one specimen per container. According to the magnesium sulfate solution concentration listed in Table 3, the corresponding magnesium sulfate solution was added to each container for that group. If the concentration listed in Table 3 was 0, pure water was added instead. The magnesium sulfate solution or pure water was required to completely submerge the specimen, and the liquid level was maintained at 5 mm above the upper surface of the specimen. Each immersion process lasted for 12 h. After immersion, the liquid in the container was removed using a micro vacuum pump (2710PW, Xinxin Electronics, Dongguan, China). In laterite-covered areas of the middle-lower reaches of the Yangtze River in China, surface temperatures can reach approximately 70 °C during summer. The drying temperature of 70 °C, which approximates the maximum surface temperature in the field, was adopted to achieve accelerated deterioration in laboratory tests. Accordingly, the specimen-holding container was placed in an oven and dried at 70 °C to constant mass. One immersion process plus one drying process constituted one wet–dry cycle.

Thereafter, the above wet–dry cycling process was repeated according to the number of cycles listed in Table 3 until all specimens reached the target number of cycles. After the environmental factor deterioration test was completed, the specimens were dried and their micro-drilling resistance was tested.

### 2.3. Method for Acquiring Micro-Drilling Resistance Curves

The DRMS main unit (SINT Technology Co., Ltd., Calenzano, Florence, Italy) and the drill bit (CYL-2, BOSCH, Gerlingen-Schillerhöhe, Germany) used for measuring the micro-drilling resistance of the stabilized laterite specimens are shown in Figure 4. This DRMS has a maximum drilling depth of 50 mm, collects drilling resistance data every 0.1 s, with a drilling speed of 1–80 mm/min, a rotation speed range of 20–1000 r/min, a maximum load of 100 N, and a resolution of 1 N. The drill bit used is a carbide drill bit with a diameter of 6 mm.

Tests were conducted on the undeteriorated SWBB-stabilized laterite specimens and those deteriorated by the combined action of multiple environmental factors as described in Section 2.2. Both dried specimens without deterioration treatment and specimens subjected to deterioration tests were placed on smooth stainless-steel gaskets during micro-drill penetration tests. A thin layer of vaseline was applied onto the upper surface of the gasket to mitigate the ring-hoop effect of the specimens. Considering the approximate strength range of the SWBB-stabilized laterite specimens, and with reference to the DRMS operating condition settings in existing research [15], the DRMS operating conditions were set as follows: drilling speed of 30 mm/min, drilling depth of 15 mm, and a constant rotation speed of 100 r/min. Three drilling runs were performed on the surface of each specimen. Three drilling points were arranged following the rules below. An equilateral triangle was projected onto the top surface of the circular specimen, with drilling points placed at its three vertices. The spacing between any two drilling points was no less than 30 mm, and the distance from each drilling point to the specimen edge was at least 10 mm. This layout takes into account potential inhomogeneity arising from specimen preparation. During the drilling process, the DRMS main unit was connected to a computer to collect micro-drilling resistance data in real time, and the drilling resistance–drilling depth curve of the micro-drill bit was recorded. After all three drilling runs for each specimen were completed, the three sets of micro-drilling resistance data were averaged to obtain the average micro-drilling resistance–drilling depth curve for that specimen.

### 2.4. Observation Method for Specimen Micro-Morphology

After the micro-drilling resistance tests were completed, the influence of different environmental factors on the micro-morphology of the SWBB-stabilized laterite was analyzed based on the test results. Representative specimens of the SWBB-stabilized laterite under specific environmental conditions were selected, and the surface micro-morphology of the specimens was observed using a scanning electron microscope (SEM). The SEM used in this study was a ZEISS Sigma 500 FE-SEM (Carl Zeiss AG, Oberkochen, Germany). When the SWBB-stabilized laterite specimens were observed, the SEM was operated in secondary electron imaging mode with an accelerating voltage of 5 kV.

## 3. Results and Discussion

### 3.1. Correspondence Between Micro-Drilling Resistance and Shear Strength of Undeteriorated Specimens

Through direct shear tests, the shear strengths of the undeteriorated stabilized specimens with curing ages of 3 d, 5 d, 7 d, 14 d, 21 d, and 28 d were obtained, as shown in Table 4.

The micro-drilling resistance–drilling depth curves of the undeteriorated stabilized laterite specimens at different curing ages obtained by DRMS are shown in Figure 5.

From the micro-drilling resistance–drilling depth curves of the SWBB-stabilized laterite at different curing ages shown in Figure 5, it can be observed that the change point between the rising stage and the stable drilling stage of the curves for undeteriorated specimens corresponds to essentially the same drilling depth. As illustrated in Figure 6, the drilling depth corresponding to the change point between the rising stage and the stable section of the drilling resistance–drilling depth curve for a undeteriorated specimen is denoted as m. Within the drilling depth range of 0 to m, the reason for the continuous increase in drilling resistance is that, as the drill bit advances from the specimen surface into the interior, the contact area between the drill bit and the specimen gradually increases, causing the micro-drilling resistance to rise continuously. After the drilling depth reaches m, the contact area between the drill bit and the specimen begins to stabilize at a constant value, and the drilling resistance value consequently remains approximately stable. Therefore, when calculating the average drilling resistance of undeteriorated specimens, the measured micro-drilling resistance values in the stable drilling stage within the drilling depth range from m to 15 mm are used for the calculation. Change point detection based on segmented regression was adopted to quantitatively locate transition depths m for each drilling resistance curve. The algorithm divided each drilling resistance curve into two segments and optimized change point positions by minimizing the sum of squared residuals.

Based on the above calculation method, the average drilling resistance of the undeteriorated SWBB-stabilized laterite specimens with curing ages of 3 d, 5 d, 7 d, 14 d, 21 d, and 28 d was calculated. The calculated average drilling resistance of the undeteriorated specimens at each curing age was linearly fitted with the shear strength of the undeteriorated specimens at the corresponding curing age. The fitted curve, prediction interval, error bars of average drilling resistance from the stable drilling stage of three drilling points for each specimen group, and the coefficient of determination are provided in Figure 7.

The results indicate that the average micro-drilling resistance of the undeteriorated SWBB-stabilized laterite specimens under either saturated or dry conditions exhibits a linear positive correlation with their shear strength. The average micro-drilling resistance serves as an indirect integrity index, which is related to the pore structure, cracking, and cementitious phases of specimens. Therefore, average micro-drilling resistance can be adopted as an indirect integrity index in subsequent tests to characterize the degree of cementation of the specimens, and further indirectly reflect their strength properties.

### 3.2. Analysis of the Deterioration Degree of SWBB-Stabilized Laterite Specimens Under Multiple Environmental Factors Based on Micro-Drilling Resistance Curves

According to the experimental scheme designed based on the Box–Behnken method in Table 3, the appearance of the SWBB-stabilized laterite specimens subjected to the combined action of multiple environmental factors is shown in Figure 8.

Micro-drilling resistance tests were conducted on the above 15 groups of stabilized laterite specimens, and Figure 9 presents the micro-drilling resistance–drilling depth curves of the 15 groups of stabilized laterite specimens deteriorated by environmental factors.

Based on the measured micro-drilling resistance values of the SWBB-stabilized laterite before and after deterioration, the influence of environmental factors on the deterioration depth and average micro-drilling resistance of the deteriorated stabilized laterite specimens was quantitatively evaluated. As shown in Figure 10, for the deteriorated specimens, the micro-drilling resistance tends to stabilize only when the drilling depth reaches n. Therefore, the difference between n and m (where m is the transition depth of undeteriorated specimens as defined in Figure 6) reflects the deterioration depth of the stabilized laterite specimens under the influence of environmental factors. The average micro-drilling resistance of the stabilized laterite specimens in the stable drilling stage under the influence of environmental factors is calculated using the measured micro-drilling resistance values within the drilling depth range from n to 15 mm.

According to the calculation procedure for deterioration depth and average micro-drilling resistance within the stable drilling stage of specimens presented in Figure 8, the average micro-drilling resistance in the stable drilling stage of specimens and deterioration depth were computed. Change point detection based on segmented regression was adopted to quantitatively locate transition depths n for each drilling resistance curve. Depth m represented the change point from the rising section to the stable drilling stage of drilling resistance for non-deteriorated specimens, whereas depth n denoted the change point marking the transition from the rising section to the stable drilling stage for deteriorated specimens. The deterioration depth was calculated as d = n − m, and the corresponding results are summarized in Table 5.

Based on the test results, it can be observed that for specimens under different combinations of environmental conditions, the deterioration depth generally increases with the increase in the number of wet–dry cycles, the initial humic acid content of the soil, and the magnesium sulfate concentration, indicating that all three influencing factors have a possible promoting effect on the increase in deterioration depth.

With the deterioration depth of SWBB-stabilized laterite after multi-factor environmental tests taken as the response value, quadratic polynomial regression fitting was performed. The regression model based on actual variable values is shown in Equation (1). In this model, A, B, and C represent the actual values of three influencing factors (wet–dry cycle number, initial humic acid content, and magnesium sulfate solution concentration), respectively. AB, AC and BC represent the interaction effects between wet–dry cycle number and initial humic acid content, between wet–dry cycle number and magnesium sulfate solution concentration, and between initial humic acid content and magnesium sulfate solution concentration, respectively. And *R* denotes the response value of deterioration depth:

(1) $$R=1.39+0.21A-0.065B+0.080C+0.020\mathrm{AB}-0.00067\mathrm{AC}+0.0013\mathrm{BC}-{0.0090A}^{2}-{0.0040B}^{2}-0.0018C^{2}$$

Analysis of variance (ANOVA) was performed on each term in the above quadratic polynomial model, and the results are shown in Table 6.

The plot of experimental versus predicted deterioration depth is presented in Figure 11.

In the quadratic polynomial regression model for the deterioration depth influenced by environmental factors, the linear effects of factors A, B, and C on the deterioration depth of the stabilized laterite are significant, and the interaction term AB is significant. This indicates that the number of wet–dry cycles, soil humic acid content, and the concentration of magnesium sulfate in the soaking solution all have linear effects on the deterioration depth of the stabilized laterite specimens. Among the factors examined, the number of wet–dry cycles exerts a comparatively significant influence on the deterioration depth of stabilized laterite specimens. Within the investigated experimental range, a statistically significant interaction exists between humic acid content and the number of wet–dry cycles with respect to deterioration depth. With the magnesium sulfate concentration fixed at its central level of 7.5 g/L, the response surface of deterioration depth against the number of wet–dry cycles and humic acid content is presented in Figure 12.

The experimental results show that when the initial humic acid content of the specimens is high, the average micro-drilling resistance in the stable drilling stage is generally low. In contrast, the average micro-drilling resistance in the stable drilling stage shows no obvious intuitive correlation with the number of wet–dry cycles or the concentration of magnesium sulfate solution.

With the average micro-drilling resistance in the stable drilling stage of the SWBB-stabilized laterite after being subjected to environmental factors taken as the response value, quadratic polynomial regression fitting was performed, yielding the quadratic polynomial regression model expressed in terms of the actual values of the variables, i.e., Equation (2), where A, B, and C represent the actual values of the three influencing factors—the number of wet–dry cycles, the initial humic acid content of laterite, and the concentration of magnesium sulfate solution, respectively; and R is the response value (average micro-drilling resistance):

(2) $$R=33.59-0.38A-0.38B-0.09C-0.06\mathrm{AB}-0.0052\mathrm{AC}-0.025\mathrm{BC}+{0.019A}^{2}+{0.012B}^{2}+0.013C^{2}$$

Analysis of variance (ANOVA) was performed on each term in the above quadratic polynomial model, and the results are shown in Table 7.

The plot of experimental versus predicted average micro-drilling resistance is presented in Figure 13.

The significance test results of the quadratic polynomial regression model for the average micro-drilling resistance in the stable drilling stage of the SWBB-stabilized laterite under the influence of environmental factors show that only factor B has a significant linear effect on the average micro-drilling resistance.

The above analysis indicates that, under the environmental conditions adopted in this study, a change point depth on the drilling resistance curve exists for the deterioration of stabilized laterite specimens induced by wet–dry cycles and magnesium sulfate soaking. Beyond this change point depth, the average stable-stage micro-drilling resistance no longer correlates significantly with the wet–dry cycle number or magnesium sulfate concentration but is predominantly governed by the initial humic acid content of laterite.

### 3.3. Micro-Morphology and Deterioration Mechanisms of SWBB-Stabilized Laterite Specimens Under the Influence of Multiple Environmental Factors

From the micro-drilling resistance test results, it can be seen that for the SWBB-stabilized laterite, the wet–dry cycles have a relatively significant influence on its deterioration depth, followed by the concentration of magnesium sulfate in the soaking solution, while the initial humic acid content of the soil and the number of wet–dry cycles exhibit an interaction effect on the deterioration depth. To further clarify the influence of the above environmental factors on the micro-morphology of the SWBB-stabilized laterite, representative combined environmental conditions as shown in Table 8 were selected. Specimens were prepared and tested under these seven environmental factor test conditions. After the tests, the surface micro-morphology of the specimens was observed using a scanning electron microscope (SEM).

Figure 14 displays the microstructural characteristics of specimens treated under the environmental test conditions given in Table 8.

For the undeteriorated SWBB-stabilized laterite specimen (D0H0M0), a dense structure was formed internally with hydration products completely encapsulating the particles. For the specimen subjected only to the influence of wet–dry cycles (D15H0M0), partial cracking occurred in the internal laterite–SWBB cementitious system, accompanied by the detachment of laterite particles. In the stabilized laterite specimen affected by both wet–dry cycles and humic acid (D15H2.5M0), the deterioration characteristics of the cementitious system around the humic acid particles were more pronounced. A weakly cemented zone composed of loose particles and pores formed between the humic acid particles and the well cemented laterite–SWBB cementitious structure, and the pore width within this zone was significantly larger than that in the specimen subjected to wet–dry cycles alone.

For the SWBB-stabilized laterite specimen subjected to the combined influence of wet–dry cycles and magnesium sulfate (D15H0M7.5), a large number of pores developed in the cementitious system, and many rod-like crystalline products morphologically consistent with ettringite precipitated in the pores; in comparison, the stabilized laterite specimen subjected to the combined influence of wet–dry cycles, humic acid, and magnesium sulfate (D15H2.5M7.5) showed a significant enlargement in pore size.

In the specimen affected only by humic acid (D0H2.5M0), a weakly cemented zone around the humic acid could also be observed; however, compared with the specimen subjected to the combined influence of wet–dry cycles and humic acid (D15H2.5M0), the development of pores within the weakly cemented zone of specimen D0H2.5M0 was less severe. Similarly, in the SEM image of the specimen subjected to the combined influence of wet–dry cycles and magnesium sulfate but with fewer wet–dry cycles (D5H0M7.5), it can be seen that the pores developed in its cementitious system were smaller in both number and size compared with the specimen also subjected to wet–dry cycles and magnesium sulfate but with more cycles (D15H0M7.5), and the amount of rod-like crystalline products precipitated in specimen D5H0M7.5 was significantly reduced.

Existing research indicates that the mechanisms by which humic acid in the soil weakens the effectiveness of the binder can be mainly classified into physical barrier effects, chemical inhibition effects, and complexation effects:

(1) The physical barrier effect refers to the adsorption of humic acid particles onto the surface of soil particles, which prevents the cementitious structure produced by the SWBB from effectively encapsulating the soil particles, thereby reducing the integrity of the stabilized soil [28];
(2) The chemical inhibition effect is that the acidic functional groups in the humic acid particles cause a decrease in pH around the humic acid particles, destroying the alkaline environment required for the hydration and cementitious reactions of the SWBB, resulting in a reduction of the hydration products generated [29];
(3) The complexation effect can be attributed to the strong chemical affinity of humic acid for calcium ions, which enables it to react chemically with the calcium ions in the SWBB to form insoluble calcium humate [30]. Calcium humate adsorbs onto the surfaces of unhydrated solid waste materials and soil particles, thereby reducing the reaction efficiency of the SWBB.

From the SEM images of the specimens deteriorated by different environmental factors listed in Table 8, it can be observed that within a certain range around the humic acid particles, the amount of hydration products increases with increasing distance from the humic acid particles, indicating that the negative effect of humic acid on the binder weakens with increasing distance. For a humic acid particle agglomerate of a certain size, the cementitious system within a distance of approximately 1–2 times the diameter of the humic acid particle agglomerate is significantly weakened.

Therefore, the SWBB-stabilized laterite containing humic acid can be regarded as a dual-porosity medium [31], with a matrix of well-cemented particles outside the influence range of humic acid particles, interspersed with weakly cemented zones composed of poorly cemented particles within the influence range of humic acid. During the wet–dry cycling process, external water infiltrates simultaneously through the preferential seepage channels constituted by the weakly cemented zones and through the tiny interparticle voids of the well-cemented regions. During the wet–dry cycling process, the accumulation and evaporation of water mainly occur in these weakly cemented zones, easily causing local pore expansion and cementitious interface detachment around the weakly cemented zones, which in turn leads to the gradual development of micro-cracks in the well-cemented portions, resulting in limited connectivity between different weakly cemented zones.

For the stabilized laterite without humic acid, during the wet–dry cycling process, the internal pores of the stabilized laterite are mainly capillary pores between different particles in the cementitious structure. During the wet–dry cycling process, water uniformly infiltrates into the soil through the capillary pores, with no obvious preferential seepage channels along the migration path. In contrast, in the stabilized laterite containing humic acid, due to the existence of weakly cemented zones, after multiple wet–dry cycles, localized preferential seepage channels will form, leading to longer water infiltration distances, and consequently a greater deterioration depth after undergoing multiple wet–dry cycles.

When the water contains solutes such as magnesium sulfate, these solutes migrate together into the interior of the stabilized laterite during the solution infiltration process. Plate-like and rod-like products resembling gypsum and ettringite were formed, and these products did not exhibit significant cementitious or binding properties. During the crystallization process, these substances exert a squeezing effect on the surrounding soil particles and the hydration products of the cemented soil particles, leading to the expansion of micro cracks, structural loosening, and an increase in porosity in the cementitious structure of the stabilized laterite [26]. The deterioration mechanisms of the above environmental factors on the stabilized laterite can effectively corroborate the significance test and ANOVA results of the environmental variables in the deterioration test of the SWBB-stabilized laterite. That is, the higher the number of wet–dry cycles, the greater the water infiltration depth and the greater the deterioration depth; the magnesium sulfate content in the soaking solution significantly affects the amount of non-cementitious substances generated at the solid–liquid interface; the weakly cemented zones around the humic acid within the stabilized laterite will gradually develop into localized preferential seepage channels with the accumulation of wet–dry cycles, accelerating water infiltration under wet–dry cycling conditions, which is manifested in the experimental results as an interaction effect between humic acid content and the number of wet–dry cycles on the deterioration depth of the stabilized laterite.

## 4. Conclusions

In this study, test conditions involving the combined action of multiple environmental factors were established. The DRMS was employed to obtain measured micro-drilling resistance–drilling depth curves of SWBB-stabilized laterite with different initial humic acid contents under wet–dry cycles and magnesium sulfate attacks. Based on the correlation between micro-drilling resistance and the shear strength of the stabilized laterite, the degree of internal deterioration of the stabilized laterite was evaluated. By combining the micro-morphology observations with the above results, the deterioration mechanisms of different environmental factors on the stabilized laterite were revealed. The main conclusions of this study are as follows:

(1) DRMS is a minimally invasive testing technique that causes little disturbance to the structure of the SWBB-stabilized laterite. The measured micro-drilling resistance–drilling depth curves of the SWBB-stabilized laterite specimens were obtained by DRMS. The correlation analysis between the shear strength of the undeteriorated SWBB-stabilized laterite specimens and their average drilling resistance values in the stable drilling stage of the micro-drilling resistance–drilling depth curves shows that the two have a linear positive correlation.
(2) Based on the measured micro-drilling resistance curves of the SWBB-stabilized laterite specimens under the influence of different environmental factors, the effects of different environmental factors on the deterioration of the stabilized laterite specimens were evaluated. The analysis results indicate that wet–dry cycles, initial soil humic acid content, and magnesium sulfate all affect the deterioration depth of the stabilized laterite specimens, and there is a synergistic effect between the number of wet–dry cycles and the humic acid content on the deterioration depth. There exists a change point in the drilling resistance curve for the effects of wet–dry cycles and magnesium sulfate on the stabilized laterite; wet–dry cycles and magnesium sulfate do not significantly affect the stabilized laterite beyond the depth corresponding to the change point.
(3) The matrix of the stabilized laterite is a densely cemented particle aggregate. The humic acid particles interspersed within it negatively affect the compactness of the cementitious structure within a range of approximately 1–2 times the diameter of the humic acid particle agglomerates, forming weakly cemented zones around the humic acid particle agglomerates. Under the action of wet–dry cycles, water forms preferential seepage channels along the weakly cemented zones, inducing pore expansion and micro-crack initiation. Meanwhile, the expansion–shrinkage stress within the soil causes spalling of the weakly cemented zones on the specimen surface, intensifying water infiltration into deeper layers. If magnesium sulfate is dissolved in the soaking solution, it will crystallize in the pores of the stabilized laterite to form poorly cemented products, making the soil structure looser.

It should be noted that this work was performed using only one type of laterite, a single binder formulation, and a fixed compaction condition, along with accelerated laboratory exposure. Nevertheless, numerous silica-dominated, iron- and aluminum-rich cohesive soils are widely distributed across tropical and subtropical regions of China. These soils exhibit similar physicomechanical properties to the laterite tested herein. Accordingly, the binder formulation proposed in this study can serve as a reference for improving the engineering performance of such soils. Furthermore, the drilling resistance measurement system established to assess the deterioration degree of stabilized soils may be cautiously extended to modification research on soils with comparable material compositions.

## Figures and Tables

**Figure 1:**
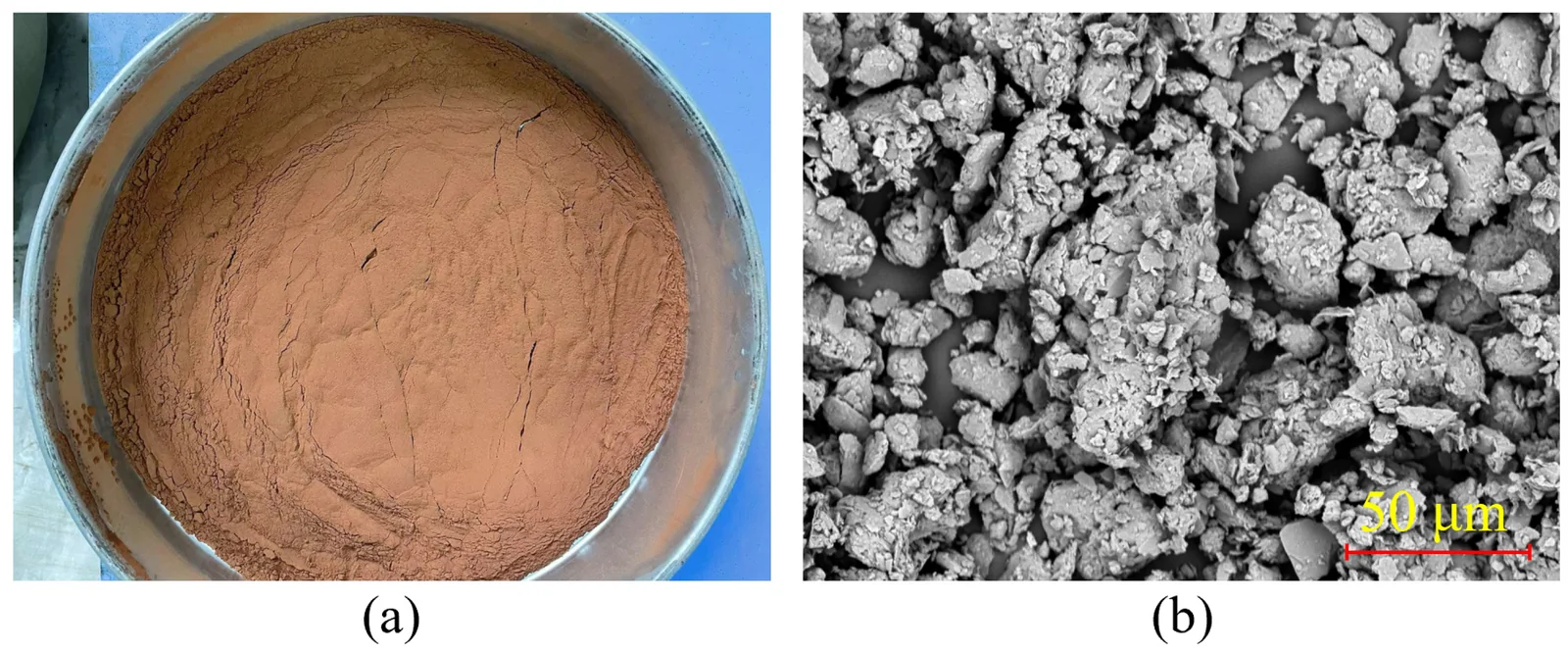
(**a**) Laterite before stabilization (**b**) Micro-morphology of the laterite.

**Figure 2:**
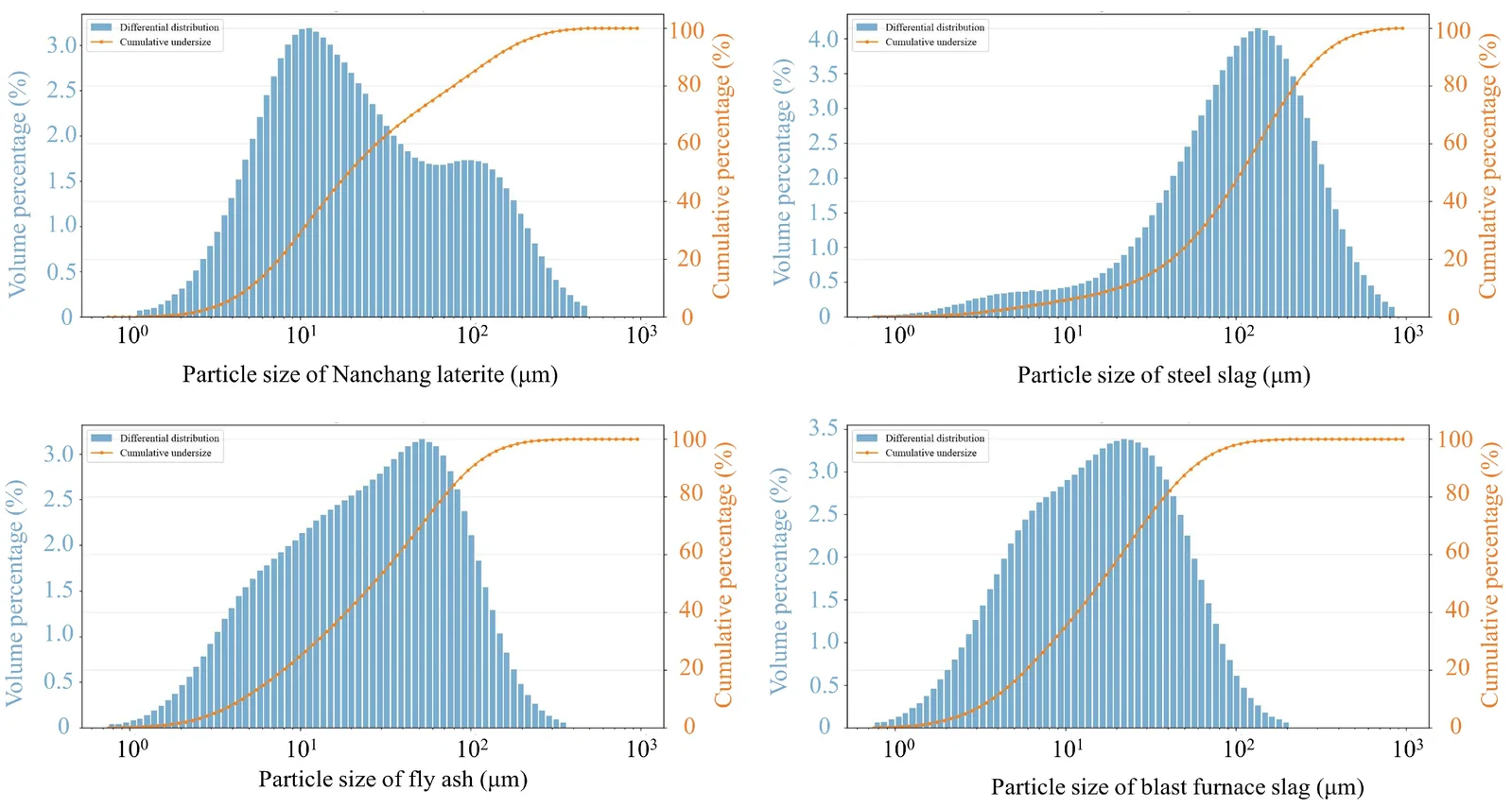
Particle size distributions of laterite, steel slag, fly ash and blast furnace slag.

**Figure 3:**
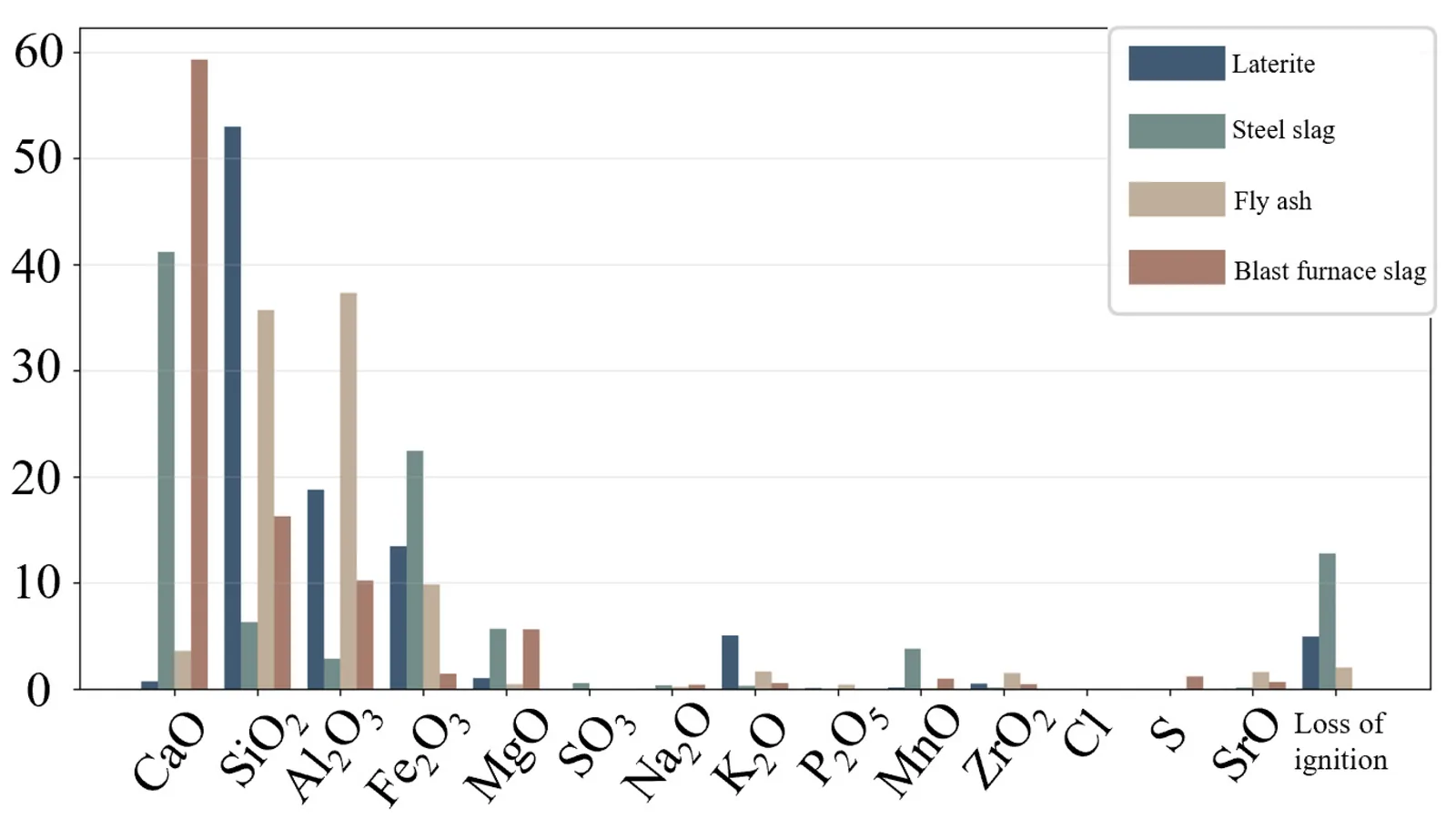
The main oxide mass fractions of laterite and three solid waste materials.

**Figure 4:**
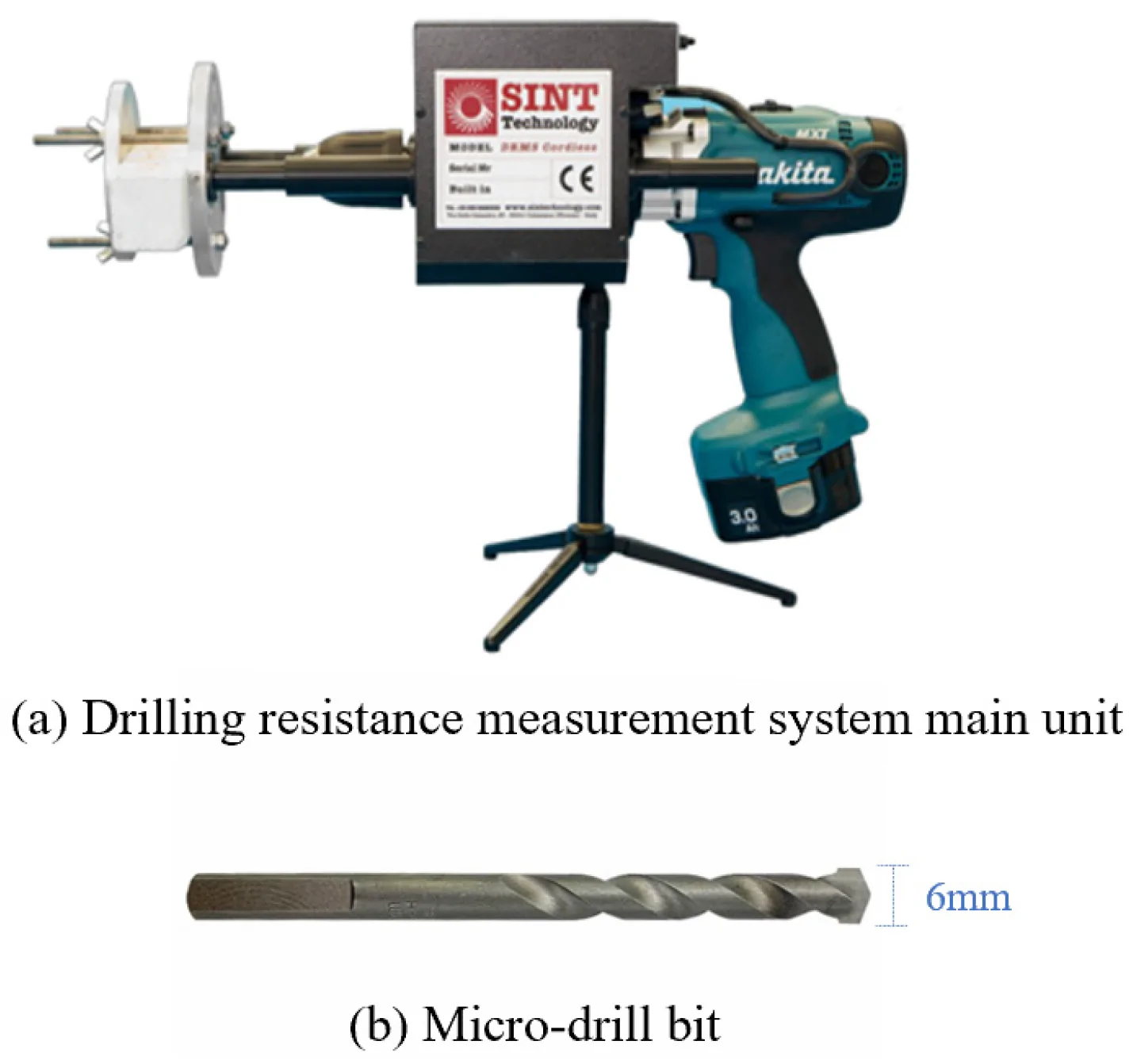
DRMS main unit and drill bit.

**Figure 5:**
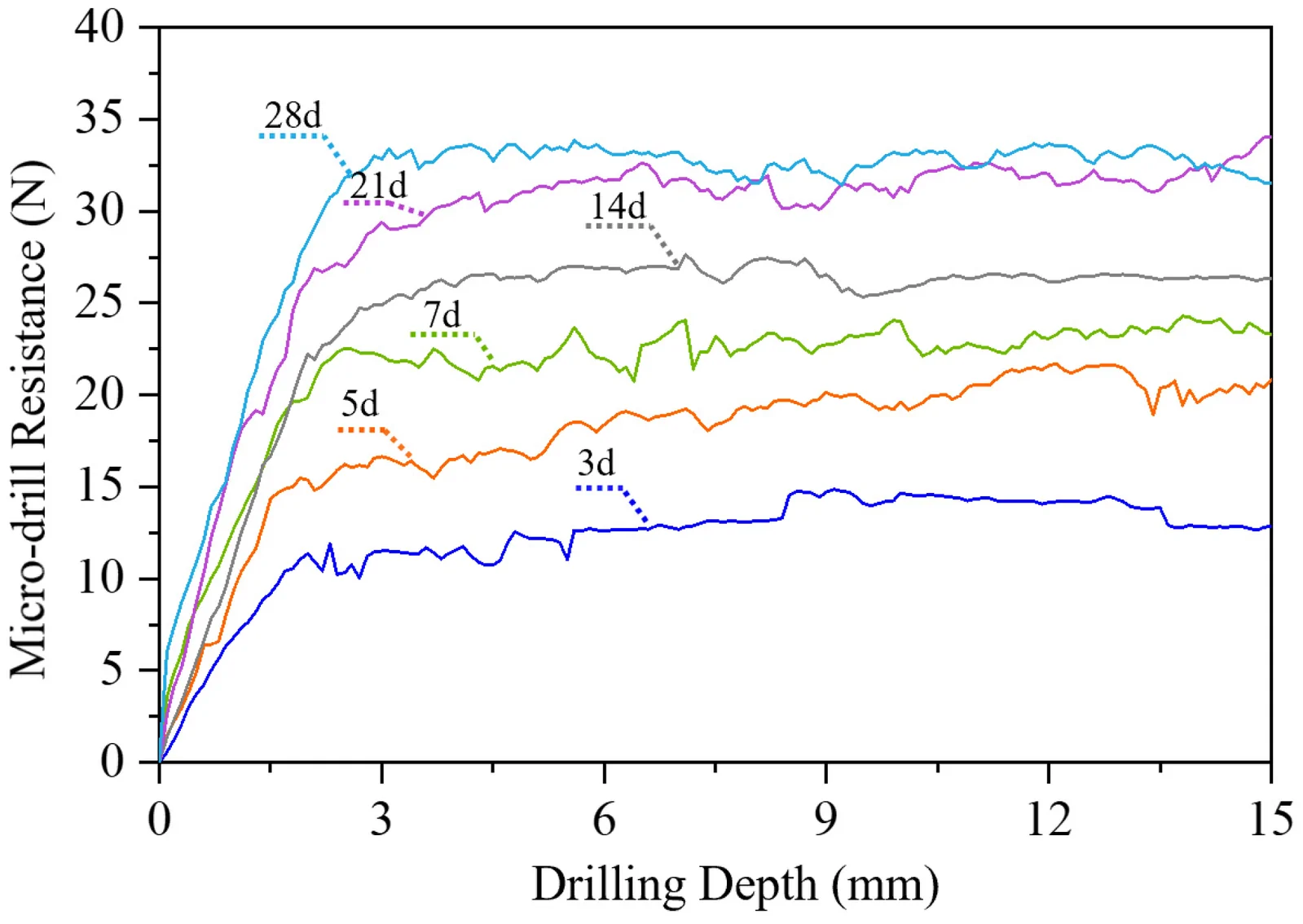
Micro-drilling resistance–drilling depth curves of undeteriorated specimens at different curing ages.

**Figure 6:**
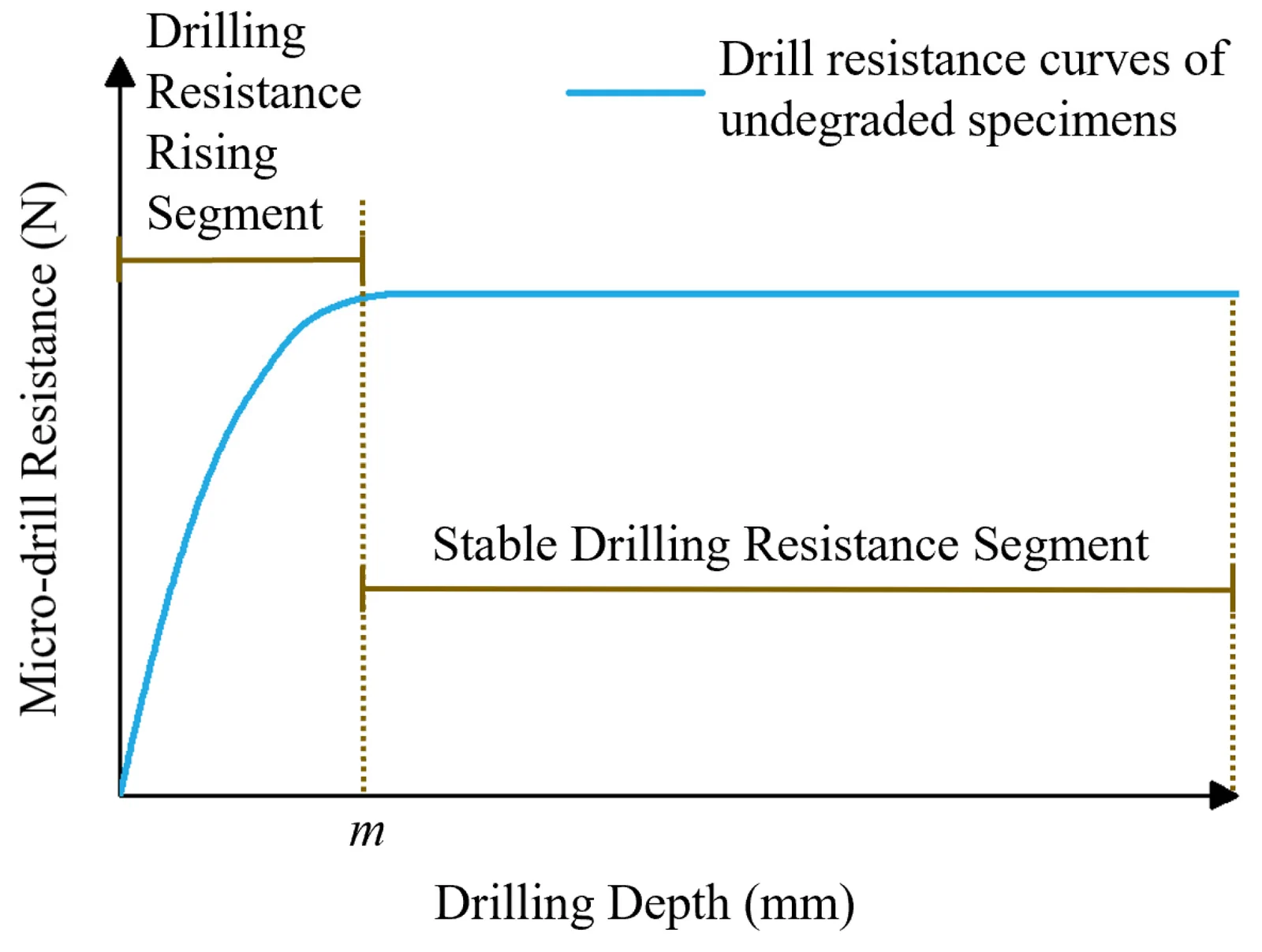
Schematic diagram of the calculation method for the average drilling resistance of undeteriorated specimens.

**Figure 7:**
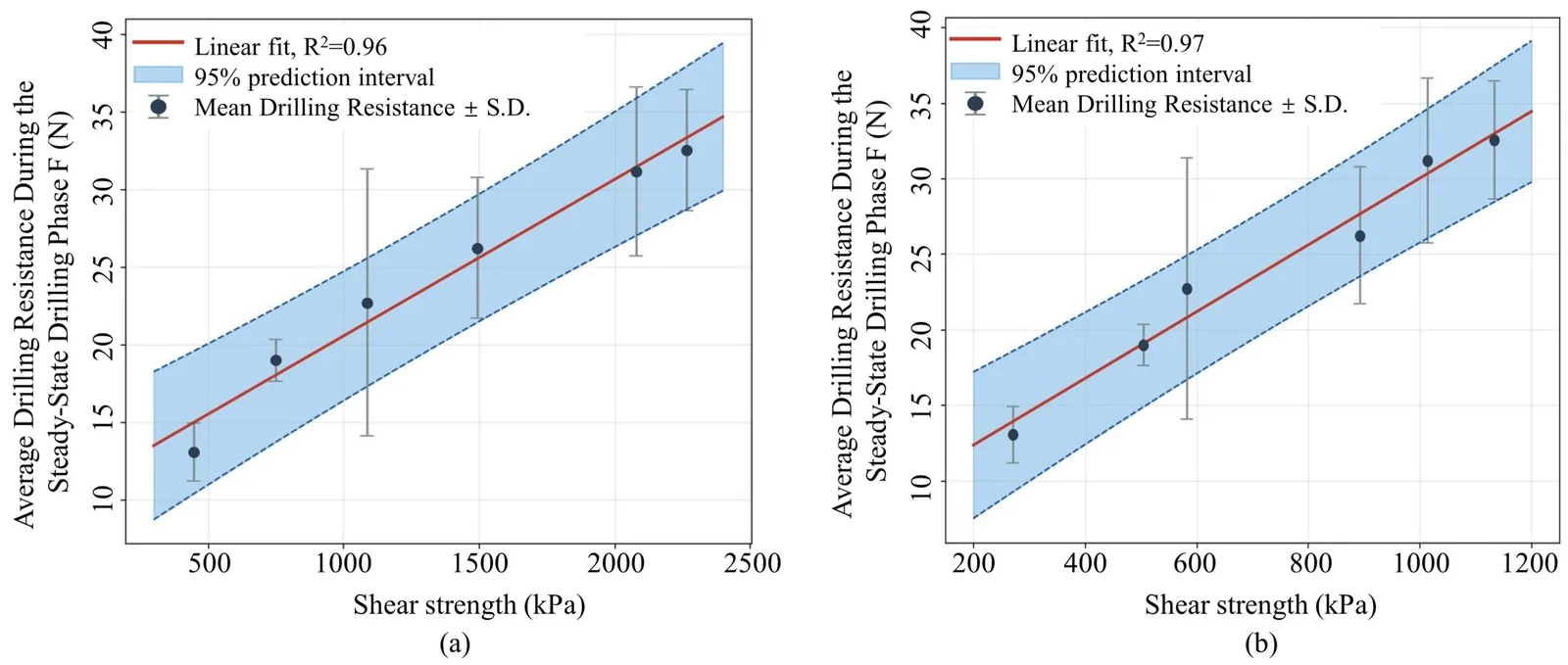
Fitting lines between average drilling resistance and shear strength of dry and saturated undeteriorated specimens: (**a**) dry specimens; (**b**) saturated specimens.

**Figure 8:**
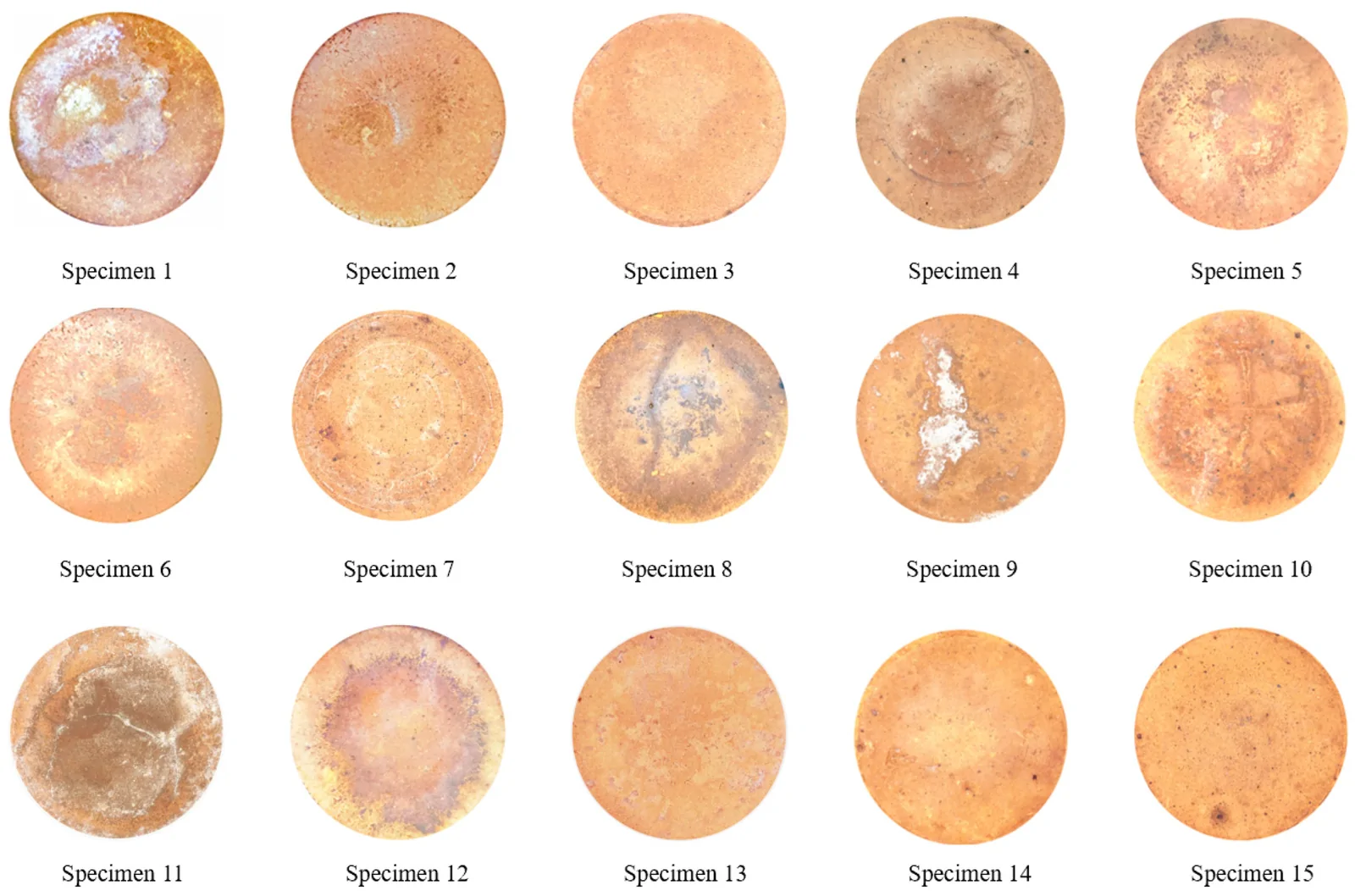
Appearance of the SWBB-stabilized laterite specimens after being subjected to the combined action of multiple environmental factors.

**Figure 9:**
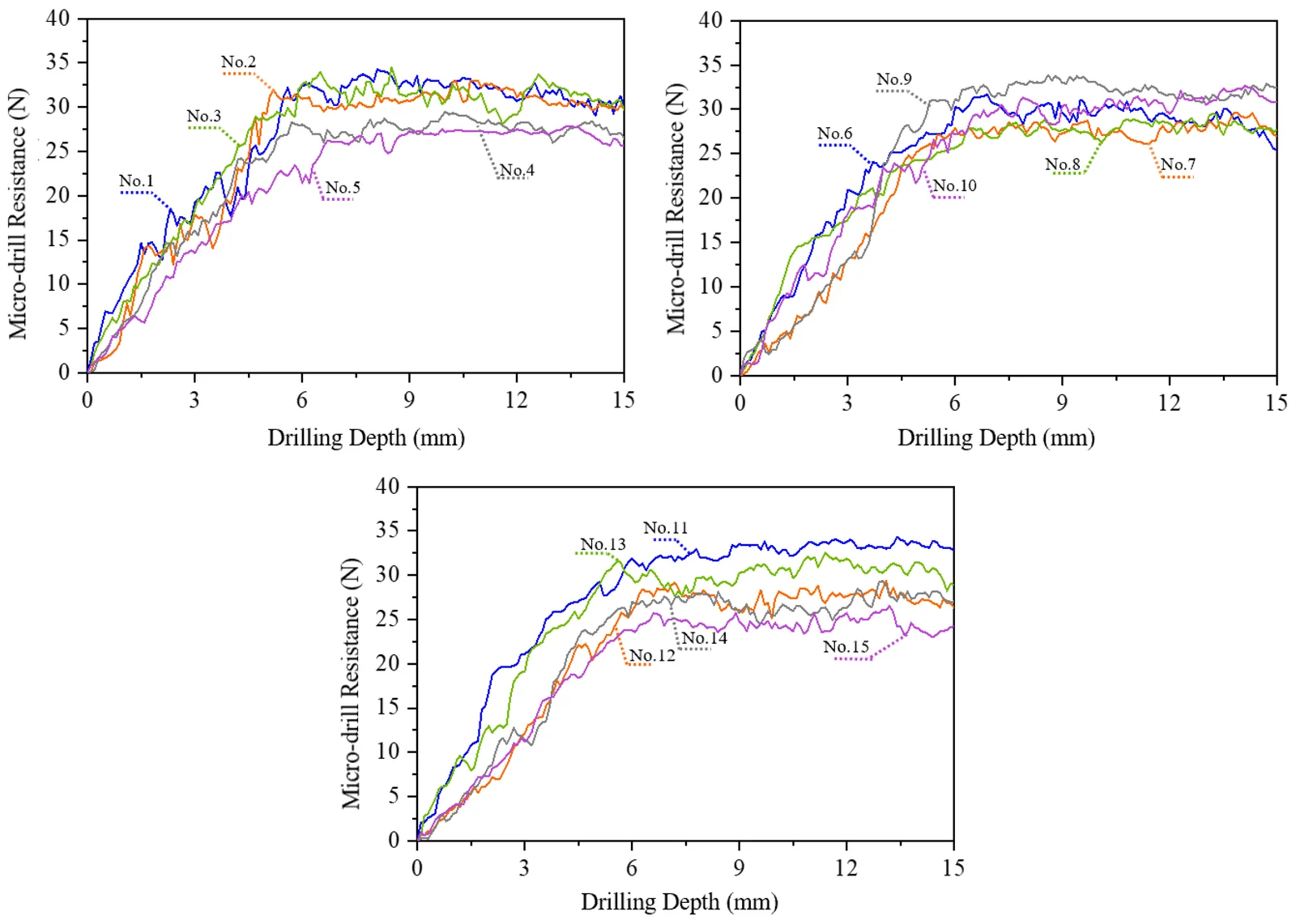
Micro-drilling resistance–drilling depth curves of the 15 groups of deteriorated stabilized laterite specimens.

**Figure 10:**
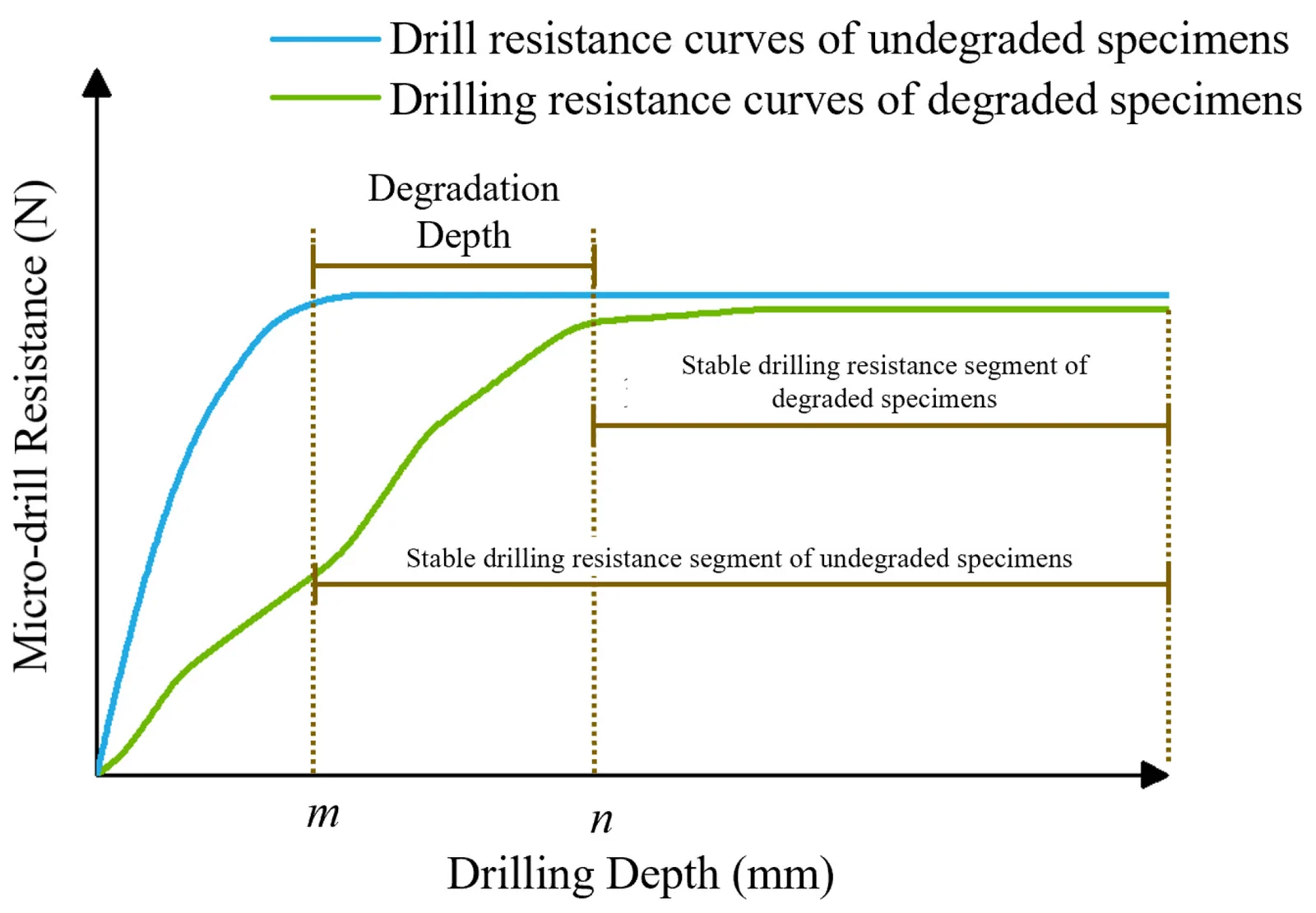
Schematic diagram of the calculation method for the deterioration depth and average micro-drilling resistance of the deteriorated specimens.

**Figure 11:**
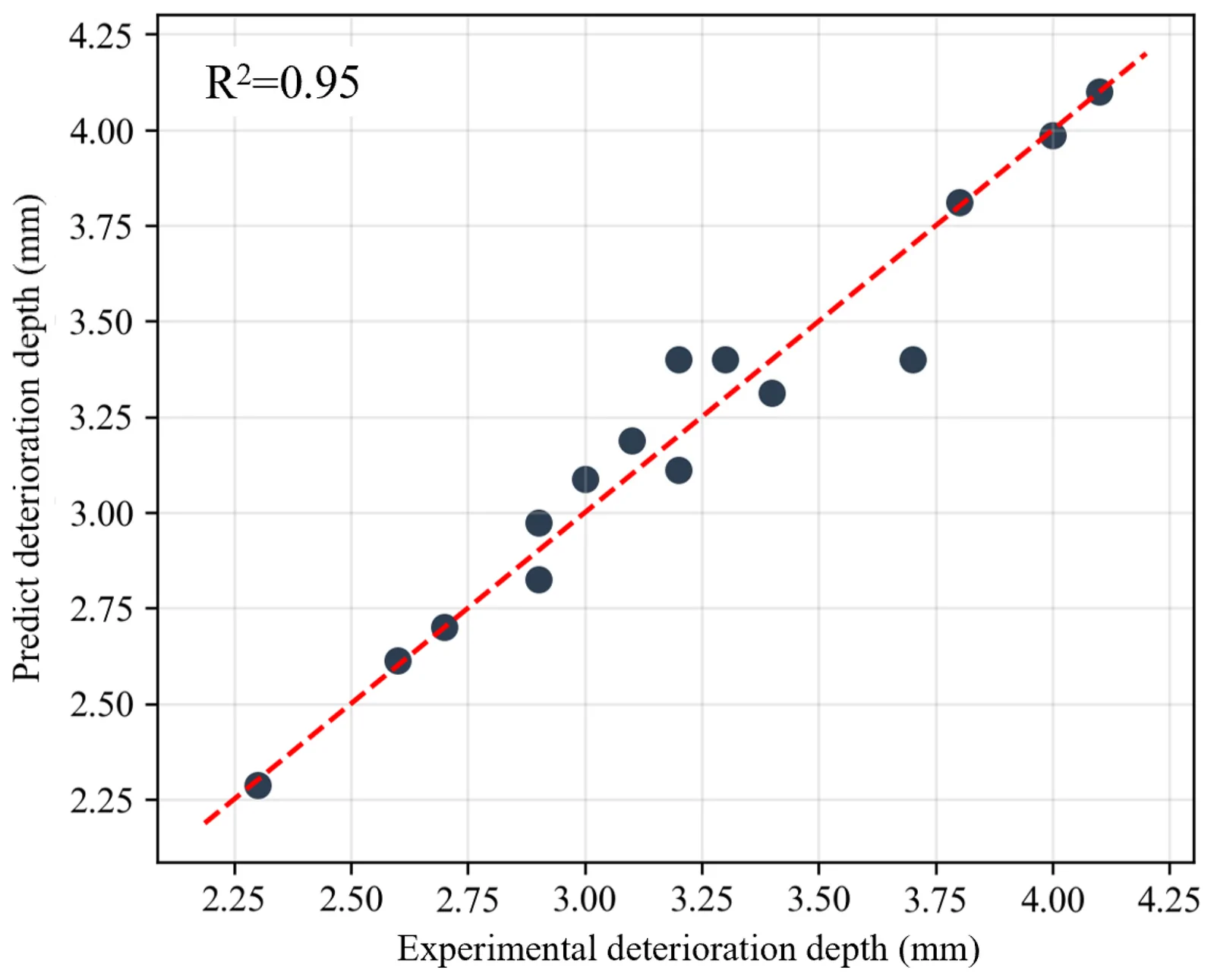
The plot of experimental versus predicted deterioration depth.

**Figure 12:**
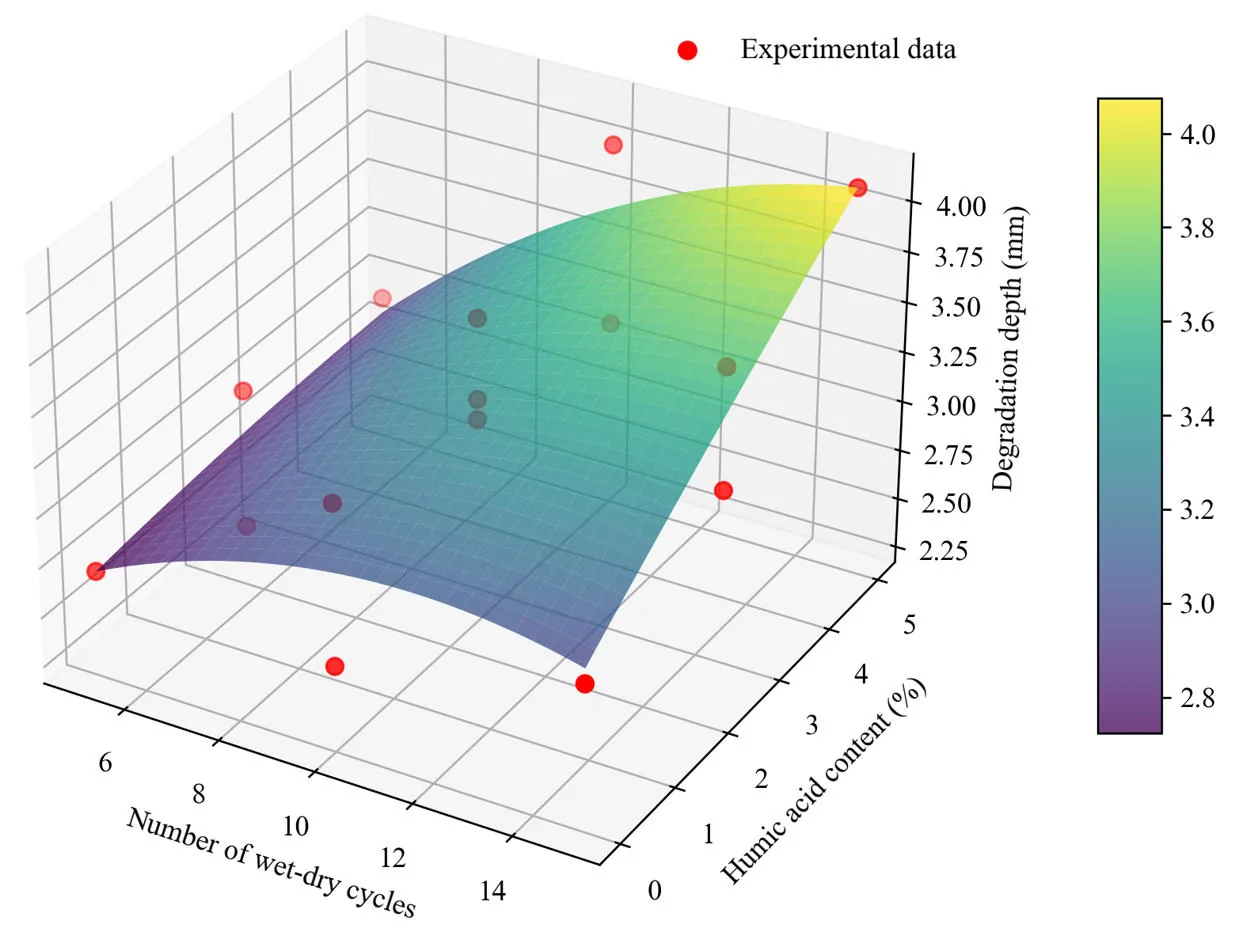
The response surface for deterioration depth versus number of wet–dry cycles and humic acid content.

**Figure 13:**
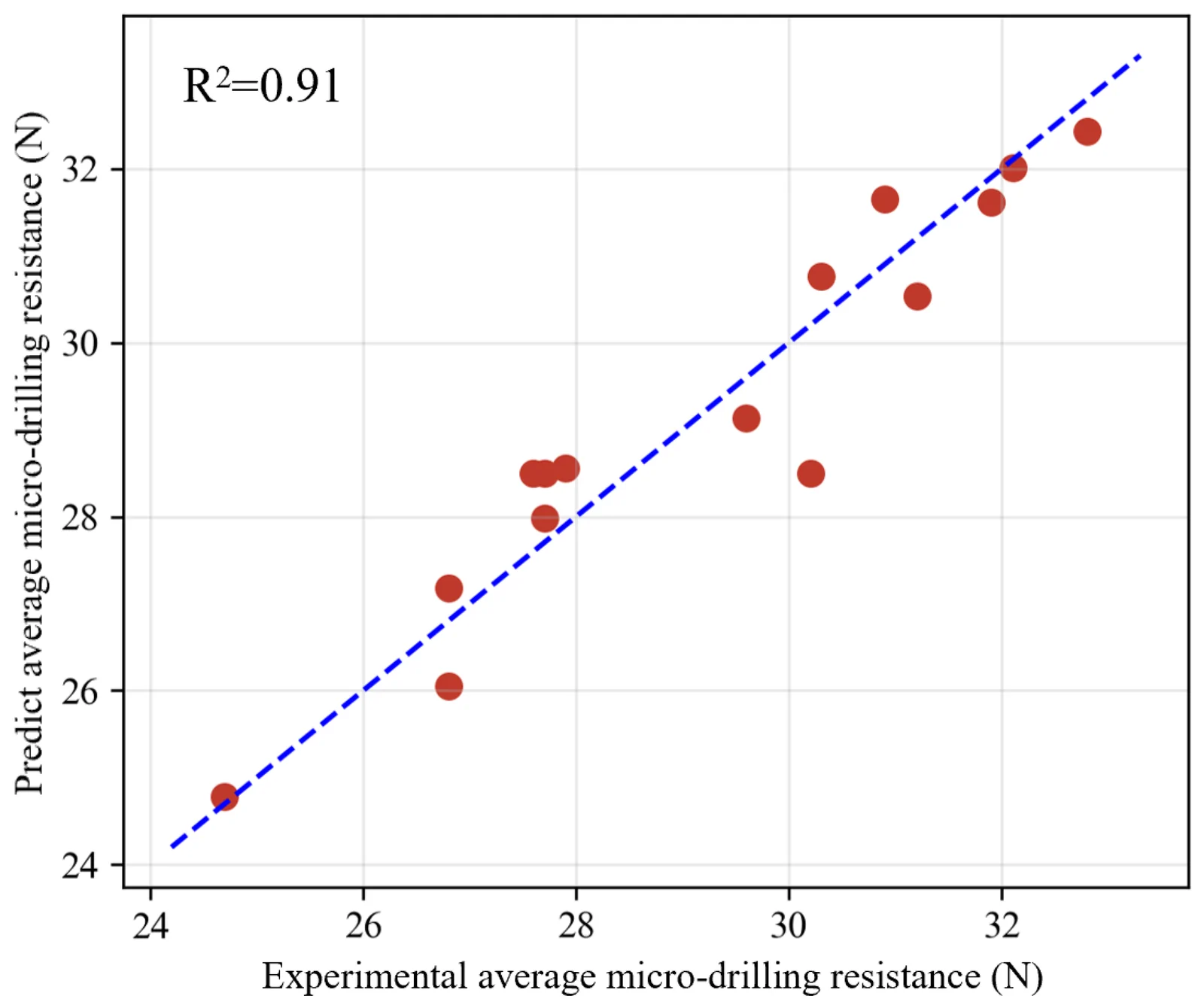
The plot of experimental versus predicted average micro-drilling resistance.

**Figure 14:**
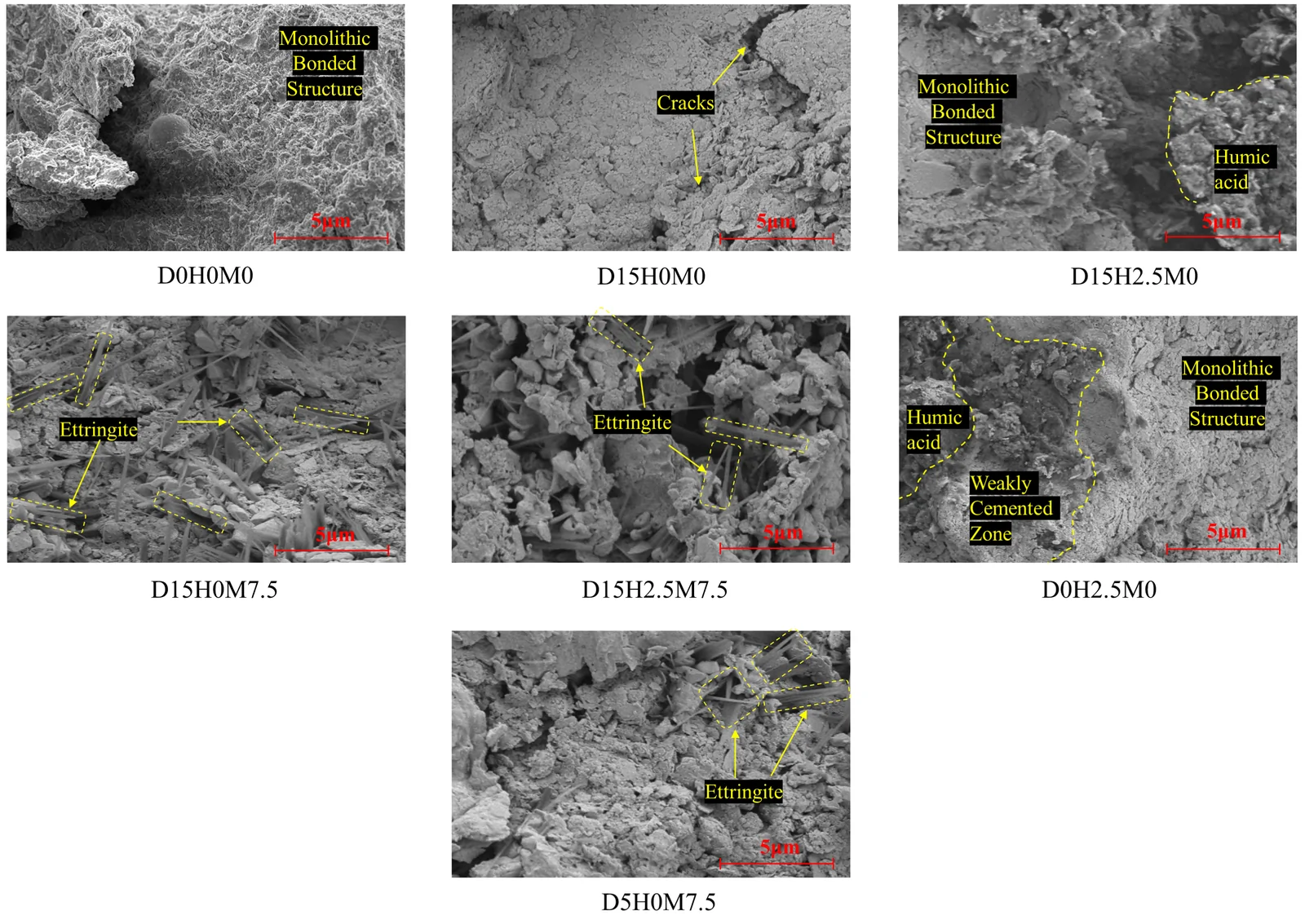
SEM images of SWBB-stabilized laterite specimens subjected to different test conditions.

**Table 1:** Cohesion and internal friction angle of laterite specimens at different moisture contents.

Moisture Content (%)	Cohesion (kPa)	Angle of Internal Friction (°)
5	14.7	35.6
10	39.4	36.1
15	56.2	37.3
20	37.6	24.6
25	3.0	21.2

**Table 2:** Actual values corresponding to the coded levels of the influencing factors.

Coding Level	Factors		
	A—Number of Dry–Wet Cycles	B—Humic Acid Content (%)	C—Magnesium Sulfate Solution Concentration (g/L)
−1	5	0	0
0	10	2.5	7.5
1	15	5	15

**Table 3:** Grouping of test conditions based on the Box–Behnken design.

Experimental Group No.	A—Number of Dry–Wet Cycles	B—Humic Acid Content (%)	C—Magnesium Sulfate Solution Concentration (g/L)
1	15	0	7.5
2	10	0	0.0
3	5	2.5	0.0
4	10	2.5	7.5
5	10	5.0	15.0
6	15	2.5	0.0
7	5	5.0	7.5
8	15	2.5	15.0
9	5	0	7.5
10	10	2.5	7.5
11	10	0	15.0
12	10	2.5	7.5
13	5	2.5	15.0
14	10	5.0	0.0
15	15	5.0	7.5

**Table 4:** Shear strength of undeteriorated SWBB-stabilized laterite specimens.

Curing Period (d)	Shear Strength of Specimens with Different Moisture Contents (kPa)	
	Dry Specimen	Saturated Specimen
3	447	271
5	750	505
7	1087	583
14	1493	892
21	2080	1014
28	2265	1133

**Table 5:** Deterioration depth and average micro-drilling resistance of the stabilized laterite specimens.

Experimental Group No.	Deterioration Depth (mm)	Average Drilling Resistance in the Stable Drilling Stage (N)	Experimental Group No.	Deterioration Depth (mm)	Average Drilling Resistance in the Stable Drilling Stage (N)
1	2.9	31.9	9	2.7	32.1
2	2.6	30.9	10	3.3	30.2
3	2.3	31.2	11	3.4	32.8
4	3.2	27.7	12	3.7	27.6
5	4.0	26.8	13	3.0	30.3
6	3.2	29.6	14	3.1	26.8
7	2.9	27.7	15	4.1	24.7
8	3.8	27.9			

**Table 6:** ANOVA of the regression model for deterioration depth.

Source	Sum of Squares	df	Mean Square	F-Value	*p* Value
Model	3.57	9	0.40	10.88	0.0086
A	1.20	1	1.20	32.91	0.0023
B	0.78	1	0.78	21.40	0.0057
C	1.12	1	1.12	30.82	0.0026
AB	0.25	1	0.25	6.85	0.0473
AC	0.0025	1	0.0025	0.068	0.8040
BC	0.0025	1	0.0025	0.068	0.8040
A^2^	0.19	1	0.19	5.12	0.0731
B^2^	0.0023	1	0.0023	0.063	0.8115
C^2^	0.037	1	0.037	1.01	0.3607
Residual	0.1825	5	0.0365		
Lack of Fit	0.0425	3	0.0142	0.2024	0.8876
Pure Error	0.1400	2	0.0700		

R-Squared = 0.95, Adjusted R-Squared = 0.86.

**Table 7:** ANOVA of the regression model for average micro-drilling resistance.

Source	Sum of Squares	df	Mean Square	F-Value	*p* Value
Model	71.41	9	7.93	5.47	0.0379
A	6.48	1	6.48	4.47	0.0882
B	59.02	1	59.02	40.70	0.0014
C	0.053	1	0.053	0.036	0.8562
AB	2.10	1	2.10	1.45	0.2824
AC	0.15	1	0.15	0.10	0.7592
BC	0.91	1	0.91	0.63	0.4637
A^2^	0.89	1	0.89	0.61	0.4693
B^2^	0.022	1	0.022	0.015	0.9059
C^2^	1.98	1	1.98	1.37	0.2949
Residual	7.25	5	1.45		
Lack of Fit	2.99	3	1.00	0.47	0.7349
Pure Error	4.26	2	2.13		

R-Squared = 0.91, Adjusted R-Squared = 0.74.

**Table 8:** Environmental factor test conditions.

Specimen Number	Curing Period (d)	Number of Dry–Wet Cycles	Humic Acid Content (%)	Magnesium Sulfate Solution Concentration (g/L)
D0H0M0	28	0	0	0
D15H0M0		15	0	0
D15H2.5M0		15	2.5	0
D15H0M7.5		15	0	7.5
D15H2.5M7.5		15	2.5	7.5
D0H2.5M0		0	2.5	0
D5H0M7.5		5	0	7.5

## Data Availability

The original contributions presented in this study are included in the article. Further inquiries can be directed to the corresponding author.

## Abbreviations

The following abbreviations are used in this manuscript:

SWBB	solid waste-based binder
DRMS	drilling resistance measurement system
RSM	response surface methodology
BBD	Box–Behnken design
